# Predictive factors regarding bullying behavior in Romanian schools

**DOI:** 10.3389/fpsyg.2024.1463981

**Published:** 2024-09-26

**Authors:** Mihaela Rus, Mariana Floricica Călin, Mihaela Luminița Sandu, Tănase Tasențe

**Affiliations:** ^1^Faculty of Law and Administrative Sciences, Ovidius University, Constanta, Romania; ^2^Institute of Psychology and Philosophy of the Romanian Academy, Bucharest, Romania; ^3^Faculty of Psychology and Educational Sciences, Ovidius University, Constanta, Romania; ^4^Faculty of Law and Administrative Sciences, Ovidius University, Constanta, Romania

**Keywords:** bullying, aggression, adolescence, school environment, conflict

## Abstract

**Introduction:**

The present study investigates the phenomenon of bullying in schools in the city of Constanta, Romania.

**Method:**

From the age point of view, we have had *n* = 210 12-year-old subjects and 193 13-year-old subjects, and from the biological gender point of view, there were 234 girls and 169 boys. The study’s main objective was to investigate aggressive behavior in adolescents in a school context. The study is cross-sectional and aims to analyze behaviors and interpersonal relationships having as dependent variable “Conflicts in the school environment” and two independent variables, respectively “Aggressive Manifestations” and “Aggressive Behaviors,” used in proving the first hypothesis, dependent variable “Verbal attacks as an aggressor” and the predictive variables “Social exclusion” and “Conflicts within the school environment” used to demonstrate the second hypothesis and the dependent variable “Bullying behavior” and the predictor variables “Acceptance of unethical behaviors,” “Violation of privacy as an aggressor” and “Dissemination of information without authorization” used to demonstrate the third hypotheses.

**Result:**

The results indicate significant correlations between aggressive behaviors and conflicts in the school environment (*r* = 0.596, *p* < 0.001), suggesting that interventions must be integrated and address the underlying causes of aggressive behaviors and associated manifestations. The association between “Conflicts in the school environment” and “Aggressive behaviors” revealed a significant correlation (*r* = 0.387, *p* < 0.001) and a moderate correlation between “Perception of conflicts” and “Aggressive manifestations” (*r* = 0.423, *p* < 0.001).

**Conclusion:**

The conclusions emphasize the importance of understanding the complexity of aggressive behavior dynamics and predictive factors for developing effective strategies for prevention and intervention in the educational environment. As a limitation of the study, it is advisable to follow the group of subjects from a longitudinal point of view to identify changes in the behavioral manifestations of these adolescents, in a school context.

## 1 Introduction

In Romania, bullying is a major problem in the school environment. According to the study carried out by the World Vision Romania Foundation (2021), approximately 46% of students reported that they were victims of bullying, while 82% of them witnessed such behaviors in their schools. The most common form of bullying identified is verbal, followed by social exclusion and physical violence. This situation is also confirmed by a study by the “Alexandru Ioan Cuza” University in Iași (2022), which shows that the prevalence of the phenomenon is high in both secondary and high schools, with a significant negative impact on mental health and academic performance of the students.

Research shows that boys are more often involved in physical bullying, while girls are more vulnerable to relational and online bullying. These forms of violence lead to serious psychological consequences, including anxiety and depression, and can contribute to lower school performance and school drop-out (World Vision Romania, 2021; Alexandru Ioan Cuza University, 2022).

In 2023, Save the Children Romania pointed out that almost 50% of students are exposed to bullying, and 4 out of 5 students have witnessed such incidents, highlighting the need for effective prevention and intervention programs (Save the Children Romania, 2023).

Order no. 6235/2023 approved by the Romanian Ministry of Education regulates the procedure for managing cases of violence against pre-schoolers, pre-schoolers, pupils, and school staff. This order sets out the steps to be followed by teachers and school management when a case of violence occurs, either inside or outside the school.

The present study mainly uses the social-cognitive theory of antisocial behavior to analyze the antisocial behaviors of adolescents in Romania. This theory was used to explore how environmental factors and cultural context influence the development and manifestation of antisocial behaviors in adolescents.

According to the social-cognitive theory, antisocial behaviors are acquired through observation and imitation in social interactions. They are influenced by the rewards and punishments that the individual perceives in his environment. This study validates the Spanish version of a self-report questionnaire of antisocial behaviors. It compares the results obtained between two distinct populations, highlighting both cultural differences and similarities in the manifestation of these behaviors (Espejo-Siles et al., 2023).

For a more detailed understanding, the authors discuss environmental influences and how changes over time and in different cultural contexts can shape antisocial behavior, thus supporting the importance of the contextual approach in the development and implementation of antisocial behavior prevention programs.

The diversity of predictive factors for bullying behaviors includes both individual characteristics, such as early aggression and behavior problems, as well as socioeconomic factors. According to the studies carried out by Jansen et al. (2011) and Hwang et al. (2017), aggression in the preschool period, low socio-economic status, and divorce represent significant elements that can lead to the involvement of adolescents in bullying behaviors.

These findings highlight the phenomenon’s complexity and indicate the need for an integrated effort in prevention and intervention.

Individual variables such as externalizing and internalizing behavior, along with contextual factors such as parental supervision and peer rejection, play a critical role in the development and extent of bullying. The studies carried out by De Sousa et al. (2021) and Fujikawa et al. (2018) emphasize the mediating importance of social skills and parenting practices in addressing these behaviors.

Research by Li et al. (2024), Fu and Zhang (2020), and Marciano et al. (2020) emphasize the importance of complex variables, from sleep problems and childhood abuse to parental psychological control and substance use, in influencing deviant behaviors, including bullying. These studies highlight the need for a holistic approach to understanding and combating bullying.

Negative childhood experiences and individual and social factors are considered predictors of bullying. Li et al. (2023) and Gilreath et al. (2022) identified a close link between bullying, sleep problems, childhood abuse, and psychosocial difficulties. Negative interactions with peers in the school environment are associated with maladaptive adjustments. These observations highlight the importance of early interventions and psychosocial support in preventing bullying behaviors.

Studies identify that gender differences and sexual orientation, along with physical and mental health factors, influence specific bullying behaviors. The study by Wang et al. (2023) indicates that adolescents with different sexual orientations face varying risks for eating disorders, which are related to bullying. Moreover, negative perceptions of the school climate contribute to the manifestation of violence, including bullying in the school context, according to Del Moral et al. (2019).

The influence of social context and personality traits on the dynamics of interpersonal relationships in bullying is significant. Research conducted by Schuetz et al. (2022) reveals that students with special educational needs are more susceptible to occupying roles as aggressors or victims. Saarento et al. (2015) emphasize that the prevalence of bullying varies based on demographic factors, group culture, and the behavior of observers, suggesting the need for an approach that includes both individual and contextual factors.

The determinants of interpersonal relationships in bullying include peer rejection, insufficient parental supervision, and deficient social skills. The study by Low et al. (2018) demonstrates a correlation between inadequate parental supervision, peer rejection, and antisocial behaviors, including bullying, highlighting the importance of a positive school environment and healthy interpersonal relationships. Tsang and Hui (2015) stress the necessity of multi-level interventions, from individual to school-wide, to effectively address bullying.

The influence of social context and personality traits on the dynamics of interpersonal relationships in bullying is significant. Research conducted by Schuetz et al. (2022) reveals that students with special educational needs are more likely to occupy bully or victim roles. Saarento et al. (2015) point out that the prevalence of bullying varies depending on demographic, cultural, and observational behavioral factors, suggesting the need for an approach that includes individual and contextual factors.

Determinants of interpersonal relationships in bullying include peer rejection, insufficient parental supervision, and poor social skills. The study by Low et al. (2018) demonstrates a correlation between inadequate parental supervision, peer rejection, and antisocial behaviors, including bullying, highlighting the importance of a positive school environment and healthy interpersonal relationships. Tsang and Hui (2015) emphasize the need for multi-level interventions, from individual to school, to effectively address bullying.

Interpersonal relationships in the context of bullying are shaped by a balance between risk and protective factors. The research of Dugre et al. (2021) identifies cannabis use and victimization experiences as key elements in differentiating behaviors. At the same time, Pereda et al. (2022) show that corporal punishment and bullying in childhood can negatively influence later relationships. These findings highlight the importance of early support and socio-emotional interventions.

Thus, the impact of victimization, empathy, and socio-emotional skills on the dynamics of interpersonal relationships in cases of bullying is very well emphasized, a fact also demonstrated by the study of Espejo-Siles et al. (2020), who show that while victimization increases the risk of violent behavior, empathy, and socio-emotional skills are activated as protective factors.

They emphasize the value of educational programs that promote the development of these skills to improve interpersonal relationships and reduce violence.

Perceptions of authority and social reputation significantly influence the complexity of interpersonal relationships in cases of bullying. Del Moral et al. (2019) note that adolescents involved in child-parental violence often display a negative attitude toward authority and aspire to a social reputation as non-conformists. This indicates an essential role of the social and school environment in the formation of aggressive behaviors and emphasizes the need for a comprehensive approach to prevent bullying.

At the same time, personal experiences and the cultural and social context play a particularly important role in the formation of attitudes and perceptions toward bullying and aggressive behaviors, as well as about the notions of morality and social responsibility. According to Kasimova et al. (2022), clinical and social factors contribute to the manifestation of suicidal behavior, highlighting a direct connection between bullying experiences and negative attitudes, such as despair and lack of coping mechanisms. In a similar study, Sitnik-Warchulska et al. (2021) acknowledge the essential role of temperament and family environment in influencing reactions to bullying and adolescents’ propensity to seek support. This indicates that intervention strategies need to address a diverse range of factors to promote beneficial attitudes and behaviors.

Emergent factors, including racial discrimination and bullying experiences, along with wider socio-cultural influences, shape adolescent attitudes and perceptions, as evidenced by research, which shows that cumulative exposure to discrimination and bullying can exacerbate socio-emotional problems and risk of obesity, thus highlighting the interconnection between mental health and bullying experiences.

The interaction between individual behaviors and social attitudes, influenced by personal experiences and cultural context, is complex. The studies of Kulis et al. (2019) and Fu et al. (2018) highlight that alcohol consumption and prosocial behavior have significant effects on cultural values and attitudes toward bullying, which demonstrates the need for a deep understanding of social and cultural dynamics to develop effective prevention programs for adolescents.

Sexual and gender minorities face unique challenges related to bullying and personal safety, as shown by studies by Reisner et al. (2014) and Taliaferro et al. (2019). Issues related to gender identity and sexual orientation require special attention in the development of bullying prevention strategies, emphasizing the importance of a safe and inclusive environment for all adolescents.

Gender differences play a significant role in the manifestation of violence, either in school or in relationships, as indicated by Baier et al. (2021).

This suggests that attitudes and behaviors related to bullying require a specific approach, sensitive to the context and demographic characteristics of adolescents, to effectively address the phenomenon of bullying.

## 2 Research objective and research questions

The objective of the research is to identify and analyze the specific behaviors that can be considered predictors of aggression among adolescents in the school context in Romania. This approach involves a detailed investigation of behavioral variables and how they contribute to the manifestation of bullying behavior.

The initiated study was structured around the following fundamental questions, formulated to analyze the phenomenon of bullying and associated aggressive manifestations in the educational environment:

### 2.1 Research question 1 (RQ1): what are the predictive factors that contribute to the emergence and intensification of specific bullying behaviors?

Bullying is a complex phenomenon influenced by a variety of factors, including individual, family, group, school, socio-economic, and cultural.

#### 2.1.1 Individual factors

Individual factors are among the most studied when discussing bullying. For example, children with an aggressive or impulsive temperament are more likely to become bullies in the school context. These children may have difficulty controlling their emotions, which makes them more likely to react violently (Smith et al., 2019). Also, lack of empathy and a thinking style based on hostility have been correlated with an increased risk of aggressive behavior (Olweus, 1993).

#### 2.1.2 Family factors

The family environment plays an essential role in the development of bullying behaviors. An authoritarian parenting style, lack of affection, or exposure to domestic violence are factors that can contribute to these behaviors. Studies have shown that children who are raised in a dysfunctional family environment, where there is frequent conflict or abuse, are more likely to exhibit bullying behaviors (Lereya et al., 2013). On the other hand, low parental supervision and poor communication between parents and children are also predictors of bullying (Baldry and Farrington, 2000).

#### 2.1.3 Group and school factors

The school environment and group dynamics play a significant role in the propagation of bullying. A school culture that tolerates violence or a lack of appropriate intervention by school personnel can intensify these behaviors. Additionally, belonging to a social group where bullying is seen as a way to gain status or power may encourage children to adopt these behaviors in order to fit in (Salmivalli, 2010). Also, peer pressure and social norms supporting aggression are significant risk factors (Espelage and Swearer, 2003).

#### 2.1.4 Socio-economic and cultural factors

Low socio-economic status and social marginalization are also factors that can contribute to bullying. Children who come from low-income families or who belong to minority groups can become targets of bullying, but at the same time, they can develop aggressive behaviors as a form of defensive reaction (Hong and Espelage, 2012). The cultural context, including community values and norms, influences the perception and acceptability of bullying behaviors (Gini and Pozzoli, 2009).

### 2.2 GAP literature

Socio-economic and cultural factors are often considered among the least studied compared to individual, family, and group factors. Although there is research that explores the impact of socioeconomic status and cultural context on bullying, it is not as numerous or detailed as studies that look at individual psychological aspects, family dynamics, or school environment influences.

More specifically, studies of how cultural norms and community practices influence bullying or how socioeconomic factors contribute to vulnerability or aggressive behaviors are less frequent. Most research focuses on specific socio-cultural environments and does not provide a global overview. Also, the impact of cultural differences on the perception of bullying and the effectiveness of interventions is an area that needs more attention.

### 2.3 Research question 2 (RQ2): are social exclusion and conflicts in the school environment predictors of bullying behavior?

Social exclusion and conflicts in the school environment are important predictors of bullying behaviors. These aspects highlight the need for a complex intervention that addresses not only individual behaviors but also group dynamics and the school climate as a whole. Promoting an inclusive school environment and effective conflict management are essential to reducing bullying and improving student well-being.

#### 2.3.1 Social exclusion

Social exclusion is a major factor that can favor the emergence of bullying behaviors. Studies show that students who are excluded or marginalized in peer groups are more likely to be victims of bullying, but may also become bullies as a way to gain power or social acceptance (Twenge et al., 2007). Feeling isolated and lacking social support in the school environment creates a fertile ground for the development of aggression, as students may seek to assert control negatively by bullying others (Bukowski and Sippola, 2001).

In addition, social exclusion can reinforce bullying behaviors, especially when peer groups encourage or tolerate such attitudes. Excluded students are often perceived as different or not conforming to group norms, making them easy targets for bullying (Nansel et al., 2001).

#### 2.3.2 Conflicts in the school environment

Frequent conflicts in the school environment, whether between students or between students and teachers, are strong predictors of bullying behaviors. Research suggests that a school climate characterized by unresolved conflict, tension, and violence increases the likelihood that students will resort to bullying as a way to manage these conflicts or express frustration (Swearer et al., 2010).

Constant interpersonal conflicts can create a hostile school environment where bullying behaviors are seen as a solution to gain superiority or cope with social pressures. Studies show that when students are frequently exposed to conflict, either as witnesses or participants, it can normalize aggression and reduce empathy for victims (Espelage and Swearer, 2004).

#### 2.3.3 The interaction between social exclusion and school conflicts

Social exclusion and conflict in the school environment do not operate in isolation. In fact, these two phenomena can influence each other, increasing the likelihood of bullying behaviors. For example, social exclusion can lead to frustration and resentment, which, when combined with a conflictual school environment, can quickly escalate into bullying behaviors (Juvonen and Graham, 2014). At the same time, a student involved in frequent conflicts may be marginalized by his peers, which may amplify the desire to reaffirm his status through acts of bullying.

### 2.4 GAP literature

Although there are studies that examine bullying in various cultural contexts, how specific cultural norms influence the relationship between social exclusion and bullying behaviors has not been sufficiently investigated. Research could examine in more detail how cultural values, such as individualism or collectivism, affect both the perception and prevalence of social exclusion and bullying.

The impact of social exclusion in the digital environment, such as social media, and how this interacts with conflicts in the school environment to promote bullying, is a relatively new and underexplored field. For example, how does exclusion from online groups or group chats contribute to school bullying behaviors?

There is a lack of longitudinal studies tracking the long-term impact of social exclusion and school conflict on the development of bullying behaviors and on the lives of adults who have been either bullies or victims. Further research in this area could provide essential information about long-term bullying prevention.

### 2.5 Research question 3 (RQ3): how is tolerance toward unethical behaviors and the publication of unauthorized information related to bullying behaviors among students?

The literature suggests that the tolerance of unethical behaviors and the publication of unauthorized information play a significant role in the increase of bullying behaviors in schools. Understanding these relationships is essential for developing effective prevention and intervention strategies that address not only bullying but also the moral and ethical norms of the school community.

#### 2.5.1 Tolerance of unethical behaviors

Tolerance of unethical behaviors such as lying, betrayal of trust or manipulation can create an environment where bullying is more likely to occur and persist. When students perceive that such behaviors are acceptable or overlooked by teachers and peers, the moral norms that discourage aggression become eroded.

According to research, in an environment where unethical behaviors are tolerated, students may become more likely to resort to bullying to gain social advantages or to strengthen their status (Rigby and Slee, 2008).

Studies suggest that when group moral norms are weak, students who might have moral qualms about bullying are encouraged to participate in or tolerate such behaviors (Thornberg, 2010). For example, if students see that minor moral transgressions are not sanctioned, they may perceive bullying as an extension of accepted behavior.

#### 2.5.2 Publication of unauthorized information

Publishing unauthorized information, especially in the digital context, is a critical aspect of modern bullying. When students share their peers’ personal information, images or messages without permission, this behavior not only violates privacy, but can lead to public humiliation and social isolation for the victims. These actions are often considered a form of cyberbullying, which has become an increasingly serious problem in contemporary schools (Kowalski et al., 2014).

Research indicates that students who engage in unauthorized posting are not only bullies, but also potential victims, as such behaviors create a cycle of revenge and retaliation. Tolerance of such actions in the school environment can amplify bullying as students learn that they can harm others without suffering serious consequences (Hinduja and Patchin, 2010).

#### 2.5.3 The interaction between tolerance of unethical behaviors and the publication of unauthorized information

Tolerance of unethical behavior and the publication of unauthorized information are often interconnected and can feed into each other in the context of bullying. For example, in an environment where unethical behaviors are tolerated, students may feel free to share unauthorized information without fear of repercussions. At the same time, success in achieving a positive social reaction by sharing compromising information can further reinforce unethical norms (Tokunaga, 2010).

### 2.6 GAP literature

Although the link between tolerance toward unethical behaviors and bullying is recognized, the specific psychological mechanisms through which these two phenomena influence each other have not been sufficiently researched. In particular, it would be important to investigate how students’ perceptions of the group’s moral and ethical norms influence their decisions to engage in bullying.

Limited longitudinal research examines the long-term effects of tolerance of unethical behaviors and involvement in whistleblowing on students’ psychosocial development. Studies could explore how these experiences influence individuals’ behaviors and ethical values in adulthood.

Little has been studied about how tolerance of unethical behavior and the publication of unauthorized information varies by cultural context. It would be interesting to explore how different cultural norms influence students’ perception and reaction to these behaviors.

Despite the increased attention to cyberbullying, there is a lack of research on the legal and ethical consequences of publishing unauthorized information in the school environment. A more detailed exploration of how school legislation and policy addresses these issues and the effect they have on student behavior would be useful.

Based on the analysis of the specialized release, the following hypotheses were proposed:

Hypothesis 1 (*H1*): The existence of correlations between aggressive behavioral manifestations determines the emergence of bullying behaviors in the Romanian educational environment.

Hypothesis 2 (*H2*): The existence of relationships between social exclusion and conflicts in the school environment are predictors of verbal bullying behavior in students acting as aggressors.

Hypothesis 3 (*H3*): The existence of correlations between tolerance toward unethical behaviors and the publication of unauthorized information/violation of privacy are predictors of bullying behavior in students.

## 3 Materials and methods

### 3.1 Participants

The analysis of gender distribution within the studied sample indicates a preponderance of female participants, they represent 58.1% (*n* = 234) of the total subjects (*n* = 403). The percentage of male participants is 41.9% (*n* = 169). Valid percentages, which exclude missing cases from the calculation, maintain the same distribution, thus illustrating a balanced composition of the sample, with a slight overrepresentation of women. Cumulatively, valid percentages reach the 100% threshold, indicating that all participants were classified into one of the two gender categories, with no cases omitted or unclassified.

### 3.2 Instruments

The applied tool aims to evaluate the manifestations of bullying in the educational context. Its methodology is based on a questionnaire structured around Likert-type questions, offering five response options, and includes a total of 55 items grouped into three scales: Bullying behaviors and interpersonal relationships (14 items, with a Cronbach’s alpha coefficient of 0.833); Evaluation of behaviors related to bullying (22 items, with a Cronbach’s alpha coefficient of 0.894); Evaluation of attitudes and perceptions related to aggressive behavior (19 items, with a Cronbach’s alpha coefficient of 0.859).

For better structuring and understanding, each scale was subdivided into subscales by applying exploratory factor analysis, using the Varimax technique for optimization, and assessing the internal consistency of each subscale.

Thus, the Bullying Behaviors and Interpersonal Relations Scale was segmented into three distinct subscales: Conflicts in the school environment (five items, internal consistency of 0.739); Aggressive Behaviors (five items, internal consistency of 0.647, indicating a relatively low value) and Aggressive Manifestations (four items, internal consistency of 0.751).

The Bullying-related behavior assessment scale includes five subscales: Violation of privacy as an aggressor (five elements, with an internal consistency coefficient of 0.872); Social exclusion (six items, with a Cronbach’s alpha coefficient of 0.809); Verbal attacks as an aggressor (five items, internal consistency of 0.759).

Publishing information without authorization (three items, internal consistency of 0.747); Violation of privacy as a victim (three elements, internal consistency of 0.751).

Finally, the scale Evaluation of attitudes and perceptions related to aggressive behaviors is divided into three subscales: Aggression/violence (seven items, with a Cronbach’s alpha coefficient of 0.812); Perception/attitude toward bullying (seven items, Cronbach’s alpha coefficient of 0.733); Tolerance of unethical behaviors (five items, internal consistency of 0.674).

This detailed structuring facilitates a deeper understanding and a more rigorous analysis of the phenomenon of bullying in educational settings.

### 3.3 Procedure

For the implementation of the research, permission was obtained from the management of the schools that were part of our study. The questionnaire was completed both physically, by the students, and through the Google Forms platform, the students had access to the questionnaire through a link that was sent to them with the help of the class leader. Thus, non-probabilistic methods were used through the convenience samples method as well as through the quota method (maintaining an approximately equal proportion for the biological gender variable) to recruit students from the 5th and 6th grades, respectively, from different schools.

### 3.4 Data analysis

Dependent variables identified in the present research include conflicts in the educational context, verbal attacks, and bullying behavior.

The predictive elements that contribute to the phenomenon of bullying are represented by aggressive behaviors, conflict in the school environment, the phenomenon of social marginalization, as well as tolerance toward unethical behavior, the violation of the right to privacy in the position of the aggressor, and the dissemination of information without the explicit consent of the targeted persons.

The data analysis process was carried out through a set of statistical procedures, ranging from elementary to the most complex methods, applied specifically for each variable, to measure characteristic descriptive parameters.

To evaluate the degree of interdependence between the studied variables, the method of correlation analysis was used. In parallel, confirmatory factor analysis was used to identify the significant predictive factors influencing and shaping bullying behavior.

The variability of one variable about another was examined using ANOVA (analysis of variance), while the regression model was used to estimate the values of one variable according to another variable.

## 4 Results

Hypothesis 1 (*H1*): The existence of correlations between aggressive behavioral manifestations determines the emergence of bullying behaviors in the Romanian educational environment.

Statistical examination of the collected data sets, relating to the variables “Conflicts in the school environment,” “Aggressive behaviors” and “Aggressive manifestations,” in the context of a sample of 403 subjects, reveals diversity in the distribution of values. The mean values calculated for “Conflicts in the school environment” were 8.98, with a standard deviation of 4.007, indicating a moderate dispersion of responses around the mean value. In the case of the “Aggressive Behaviors” variable, the calculated mean was 6.33, with a standard deviation of 2.052, highlighting a narrower variation in the data. Regarding the variable “Aggressive Manifestations,” the recorded mean was 7.49, with a standard deviation of 3.427, illustrating a distribution with a relatively moderate dispersion.

From a psychological perspective, these results suggest that, within the educational context, the phenomenon of “Conflicts” is perceived as having the highest frequency and variability, followed by “Aggressive Manifestations” and subsequently by “Aggressive Behaviors.”

The higher average value associated with “Conflicts in the school environment” shows a general recognition of conflicts as a notable and problematic element in the educational environment. This, coupled with a significant standard deviation, indicates that individuals’ experiences of conflict vary considerably. “Aggressive behaviors,” recording the lowest mean and standard deviation, can be interpreted as less prevalent and more consistent among the analyzed sample.

However, the responses to the “Aggressive Manifestations” scale suggest that paying more attention to how aggression manifests and is perceived in the school setting is imperative. In conclusion, these findings emphasize the need to adopt different and personalized strategies to understand and effectively intervene in the problem of conflicts and aggressive behaviors in educational institutions.

In the analysis of the Pearson correlation coefficients for the variables “Conflicts in the school environment,” “Aggressive behaviors” and “Aggressive manifestations” in a sample composed of 403 subjects, the following significant results were obtained:

- The interaction between “Conflicts in the school environment” and “Aggressive manifestations” registered a correlation coefficient of 0.596, significant at the 0.00 level, reflecting a positive correlation of moderate to high intensity.
- This significant relationship suggests a significant association between the perception of conflicts and the frequency of aggressive manifestations, indicating the possibility that environments characterized by heightened conflicts favor the emergence of aggressive behaviors.
- The association between “Conflicts in the school environment” and “Aggressive behaviors” revealed a correlation coefficient of 0.387, significant at the 0.00 level, suggesting a positive correlation, but of weak to moderate intensity.
- The moderate correlation between “Aggressive behaviors” and the other two variables (having coefficients of 0.387 and 0.423) suggests that although there is a significant connection between aggressive behaviors, conflicts, and aggressive manifestations, this interconnection is not as strong as that observed between perceptions of conflicts and aggressive manifestations. This could indicate that the variable of aggressive behaviors is influenced by a wider spectrum of factors, not only by conflict dynamics or direct aggressive manifestations.

In conclusion, these findings emphasize a significant interdependence between conflicts in the school environment, aggressive behaviors, and aggressive manifestations.

These results emphasize the need to adopt integrated and well-founded strategies within educational interventions, to reduce the level of aggression and effectively manage conflicts in educational institutions. Thus, a thorough understanding of how these variables interact and influence each other in the specific context of the educational environment is essential.

The KMO coefficient is 0.649 (Table 1), which allows us to moderately consider that these existing correlations between the studied variables are not due to chance and allows us to apply factor analysis to determine the degree of influence of conflicts in the school environment, an influence that is also determined by the aggressive behaviors manifested in the school.

**Table 1:** KMO and Bartlett’s Test.

Kaiser-Meyer-Olkin measure of sampling adequacy.		0.649
Bartlett’s test of sphericity	Approx Chi-square	268.093
	df	3
	Sig.	0.000

The model presented in Table 2 investigates the association between the dependent variable “Conflicts in the school environment” and two independent variables, namely “Aggressive Manifestations” and “Aggressive Behaviors.” The coefficient of determination, R Square (*R*^2^), recorded at the value of 0.377, illustrates the proportion of variation in the dependent variable (“Conflicts in the school environment”) that can be attributed to the influence of the combination of the two independent variables. The value of 0.377 indicates that approximately 37.7% of the dispersion of school conflicts is explained by the variability of aggressive manifestations and behaviors.

**Table 2:** Model summary–the association between the dependent variable and the independent variables.

Model	*R*	*R* square	Adjusted *R* square	Std. error of the estimate	Change statistics				
					*R* square change	*F* change	df1	df2	Sig. *F* change
1	0.614 ^a^	0.377	0.374	3.171	0.377	121,055	2	400	0.000

^a^Predictors: (Constant), Aggressive manifestations, Aggressive behaviors. ^b^Dependent Variable: Conflicts in the school environment.

From a psychological perspective, these results indicate that, although aggressive manifestations and behaviors are relevant factors in the elucidation of conflicts in the school environment, there are other factors, that represent approximately 62.3% of the variation in school conflicts, and which are not included in this model.

These factors may include elements such as the climate of the educational institution, the dynamics of interpersonal relationships, external pressures, or the individual characteristics of students.

In conclusion, the statistical model underlines a moderate but significant association between “Conflicts in the school environment” and the independent variables “Aggressive Manifestations” and “Aggressive Behaviors.”

The Adjusted R-Square coefficient attests to an adequate fit of the model, and the F-statistic analysis confirms a notable contribution of the independent variables in explaining the observed variation in the dependent variable.

The statistical evaluation presented in Table 3 reveals that the results of the analysis of variance (ANOVA) are significant (with a Sig. value = 0.00), thus highlighting the relevance of investigating and systematically addressing aggressive behaviors and manifestations within educational interventions, to mitigate conflicts. Aggression, conceptualized through the prism of manifestations and behaviors, plays a significant role in the structure and evolution of conflicts in the school context. These findings suggest that prevention and intervention programs should aim not only at effective conflict management and resolution but also at identifying and addressing predisposing factors for aggressive behaviors and manifestations. A proactive strategy could include implementing social skills education programs, organizing workshops focused on anger and frustration management, and promoting programs aimed at cultivating empathy and awareness.

**Table 3:** ANOVA analysis of variance, between the dependent variable and the predictor variable.

Model		Sum of Squares	df	Mean Square	F	Sig.
1	Regression	2434.205	2	1217.102	121,055	0.000 ^b^
	Residual	4021.636	400	10,054		
	Total	6455.841	402			

^aDependent variable: conflicts in the school environment.^ ^b^Predictors: (Constant), aggressive manifestations, aggressive behaviors.

In conclusion, the results of the ANOVA analysis indicate a strong and statistically significant association between aggressive behaviors and manifestations and the frequency of conflicts in the school environment, thus constituting a robust foundation for the development and implementation of targeted and effective strategies aimed at improving the educational climate.

Examination of the coefficients of the regression model, presented in Table 4, provides a detailed insight into the relational dynamics between the independent variables (“Aggressive Behaviors” and “Aggressive Manifestations”) and the dependent variable (“Conflicts in the school environment”). The interpretation of these data underlines the determining role of perceptions, attitudes, and social norms prevailing within educational institutions in shaping the behavior of students. In this context, aggressive manifestations can contribute to establishing a climate conducive to the development or escalation of conflicts.

**Table 4:** Coefficients–the regression coefficient between the dependent variable and the predictor variables.

Model		Unstandardized coefficients		Standardized coefficients	*t*	Sig.	Correlations			Collinearity Statistics	
		*B*	Std. error	Beta			Zero-order	Partial	Parthian	Tolerance	VIV
1	(Constant)	2,339	0.535		4,371	0.000					
	Aggressive behaviors	0.322	0.085	0.165	3,788	0.000	0.387	0.186	0.149	0.821	1,217
	Aggressive manifestations	0.615	0.051	0.526	12,078	0.000	0.596	0.517	0.477	0.821	1,217

^a^Dependent variable: conflicts in the school environment.

In the multiple regression, the potential predictors of bullying behavior were entered in the ascending order of the correlation coefficients obtained by each of them with conflicts in the school environment. The regression equation of bullying behaviors in the educational environment has the following elements:

- The Adjusted *R*-squared value of 0.374 means that the regression model explains 37.4% of the total variation in the dependent variable “conflicts in the school environment” based on the predictors included in the model. This represents a modified version of the *R*^2^ coefficient, adjusted for the number of predictors present in the model and the sample size.

The proximity of the Adjusted *R*^2^ value to the *R*^2^ value suggests that adding additional predictors to the model was relevant and appropriate, without causing significant overestimation. The remaining variation refers to the proportion of the dependent variable’s variance that is not explained by the current model.

In this case, the remaining variation is 62.6% (100–37.4%), which means there are factors or confounding variables that could influence “conflicts in the school environment” but were not included in the current regression model. These factors include personal factors, such as students’ personality traits, for example, impulsivity and empathy, family factors, including family dynamics, parenting style, and the family’s socio-economic status, cultural and socio-economic factors, including cultural norms regarding aggressive behavior and the community’s socio-economic level, the influence of the online environment and social media, such as exposure to cyberbullying and the influence of social networks, school policies and programs, referring to the effectiveness of anti-bullying programs and the school’s disciplinary policies, interpersonal interactions, including relationships with peers and teachers and social support from friends and mentors.

- *F*-test (ANOVA) values and significance coefficients having values less than 0.000 confirm that the model is valid

Knowing the level of aggressive manifestations in the educational environment and aggressive behaviors using the regression equation - *conflicts in the school environment = 2.339 + (0.322) aggressive behaviors + (0.615) aggressive manifestations* - we obtain the level of bullying behaviors in the school environment.

**Figure 1:**
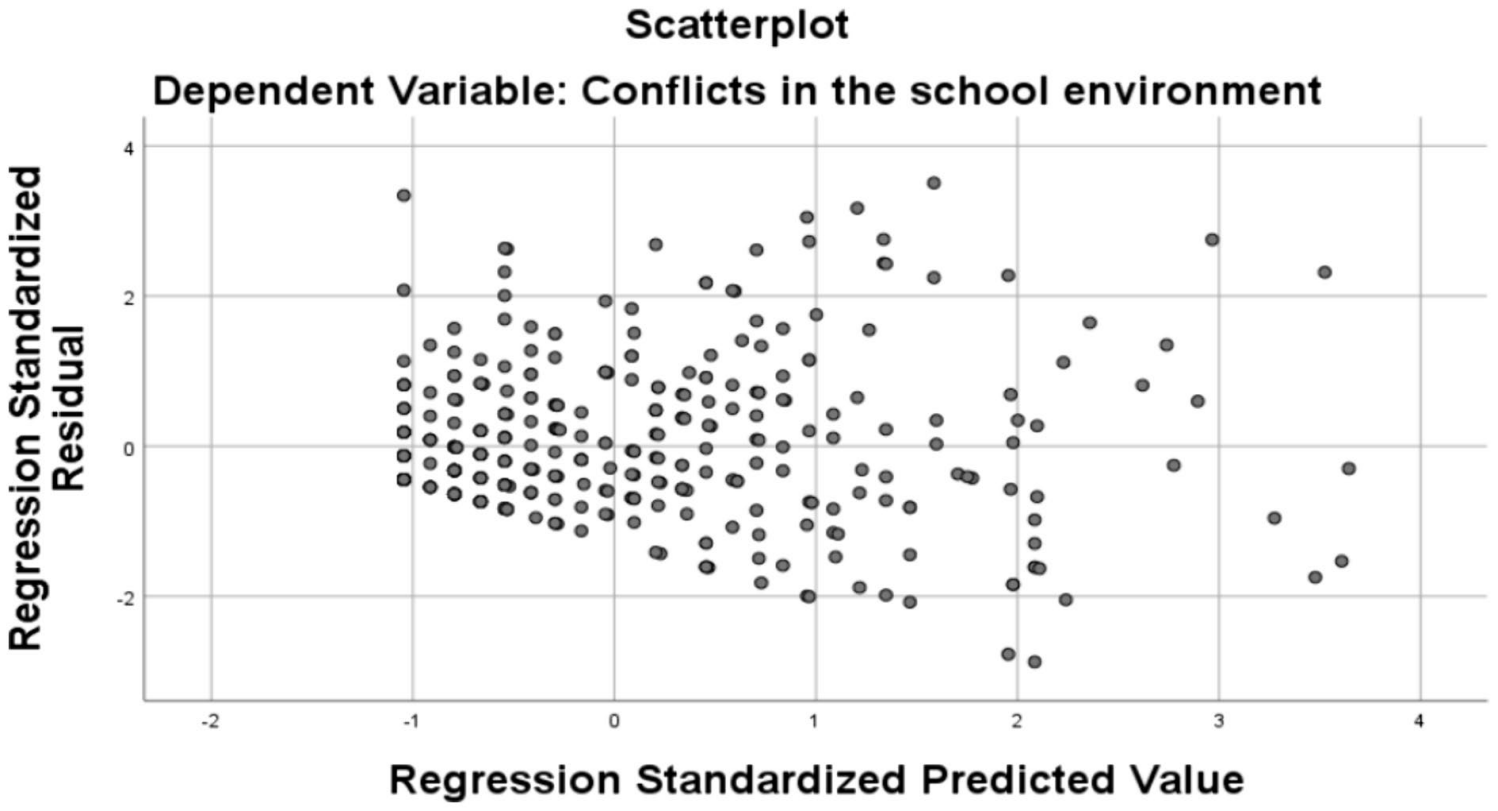
Scatterplot.

The conclusions drawn from the data analysis suggest that both behaviors and aggressive manifestations are significant predictors of the incidence of conflicts in the school environment, with a relatively stronger influence exerted by aggressive manifestations. The coefficients of the model, the level of significance, and the established correlations confirm the existence of a robust and significant interaction between these variables and the frequency of conflicts in the educational environment (Figure 1).

Examination of the scatterplot indicates that the regression model is appropriate for the data set analyzed, given that the residuals are generally evenly distributed with no evidence of heteroscedasticity or non-linearity. This infers that the regression model provides a reliable predictive estimate for the variable “Conflicts in the school environment,” except for potential extreme values (outliers) that may require further analysis. In the present case, the plot shows no pronounced patterns, suggesting that the variance of the residuals is relatively stable.

Hypothesis 2 (*H2*): The existence of relationships between social exclusion and conflicts in the school environment are predictors of verbal bullying behavior in students acting as aggressors.

The statistical analysis was performed on a data set comprising three variables: “Verbal attacks as an aggressor,” “Social exclusion” and “Conflicts in the school environment,” each with several 402 observations. For the indicator “Verbal attacks as an aggressor,” the average of 6.02 reflects a moderate incidence of this type of behavior. This level of verbal aggression could signal the existence of tensions or communicative dysfunctions among students and can be interpreted as a marker of self-control deficits or of an institutional culture that does not sufficiently discourage aggressive behavior. In the case of the “Social exclusion” indicator, the average of 9.36 is the highest among the three variables, signaling a strong presence of the phenomenon of social marginalization among the participants. A standard deviation of 4.276 indicates a significant dispersion of experiences of social exclusion, suggesting notable differences between participants in this regard. A high mean in the context of wide dispersion may reflect a widespread problem with profound adverse psychological effects, such as feelings of isolation, depression, and anxiety, affecting individuals differently.

For the indicator “Conflicts in the school environment,” the mean of 8.96 suggests that conflicts are a significant problem, with a standard deviation of 3.992, also indicating a variety of students’ experiences related to these conflict situations. This value proximity between the media for conflict and that for social exclusion emphasizes that both phenomena are frequent and relevant problems, having the potential to negatively influence both academic performance and the emotional well-being of students.

The analysis of the data suggests that the phenomenon of social exclusion and conflicts in the school environment are more prominent than verbal aggression, although all three variables are present and could be interrelated. The high variability observed for social exclusion and school conflicts indicates a significant diversity of individual experiences, providing a starting point for the development of tailored interventions.

Although this analysis provides insight into the prevalence of these behaviors, it would be prudent to consider other variables, such as educational background, family relationships, and social support, to gain a deeper understanding of the causal factors and dynamics of these problems.

The correlational study examined the relationships between the variables “Verbal attacks as an aggressor,” “Social exclusion” and “Conflicts in the school environment,” using the Pearson correlation coefficient and evaluating the statistical significance of these correlations for several 402 observations for each variable.

The analysis revealed a positive correlation of moderate intensity (0.581) between the frequency of verbal attacks and the incidence of social exclusion, indicating a trend of simultaneous growth of these phenomena. This association suggests a potential interdependence between verbally aggressive behavior and social marginalization within the study population.

The relationship between “Verbal attacks as an aggressor” and “Conflicts in the school environment” was identified as positive, but of lower intensity (0.329), signaling a less obvious connection between verbal aggression and school conflicts. This suggests that although there is an association between the two variables, other factors may contribute significantly to the dynamics of conflict in the educational environment.

The correlation between “Social Exclusion” and “School Conflicts” (0.585) is comparable to that between verbal attacks and social exclusion, illustrating a moderate association. This correlation underlines a possible significant relationship between experiences of social exclusion and involvement in school conflicts, suggesting that marginalized students may be more susceptible to participating in conflicts or, conversely, conflicts may facilitate exclusion phenomena.

The statistical significance of all correlations at the 0.000 level (one-tailed) confirms the improbability of these relationships being the product of randomness, emphasizing the significant interdependence between verbal attacks, social exclusion, and school conflicts. These findings emphasize not only the coexistence of these behaviors but also the possibility of mutual influence.

In the context of educational interventions, it is essential to recognize the interconnected nature of these issues and to address common underlying factors such as institutional climate, social–emotional skills, and student support. The results emphasize the need for a holistic approach to managing school problems, rather than an exclusive focus on a single type of disruptive behavior.

Since the KMO coefficient is 0.603 (Table 5), we can moderately consider that these existing correlations between the studied variables are not due to chance, which allows us to apply factor analysis to determine the percentage of influence of verbal attacks exerted by the aggressor based on conflicts in the school environment and the social exclusion they experience.

**Table 5:** KMO and Bartlett’s test.

Kaiser-Meyer-Olkin measure of sampling adequacy		0.603
Bartlett’s test of sphericity	Approx. Chi-Square	331.546
	df	3
	Sig.	0.000

The study of the relationship between the predictor variables (“Social exclusion” and “Conflicts in the school environment”) and the dependent variable (“Verbal attacks as an aggressor”), as presented in Table 6, requires a detailed analysis of each element of the model summary, addressing both statistical aspects and psychological implications. The multiple correlation coefficient (*R* = 0.581) denotes a correlation of moderate intensity between the predictor variables and the dependent variable, indicating that the level of social exclusion and the frequency of school conflicts have a moderate association with the prevalence of verbal attacks as a form of aggression. From a psychological perspective, this moderate correlation suggests that the social context and conflict situations in which students find themselves contribute significantly to the adoption of verbally aggressive behavior, possibly reflecting a self-defense mechanism or a means of expressing accumulated frustrations. The coefficient of determination (*R* Square = 0.337) illustrates that approximately 33.7% of the variance of verbal attacks as an aggressor can be attributed to the influence of the combination of the predictor variables of social exclusion and school conflicts. The Adjusted *R*-Square index (0.334), which adjusts the *R*-Square for the number of predictors in the model and the amount of data, provides a more accurate assessment of the model’s predictive ability in the sample. The closeness of the Adjusted *R* Square value to *R* Square reaffirms the fit of the model and suggests that it is not over-fitted.

**Table 6:** Model summary^b^ the association between the dependent variable and the predictor variables.

Model	*R*	*R* Square	Adjusted *R* square	Std. error of the estimate	Change statistics				
					R square change	F change	df1	df2	Sig. *F* change
1	0.581 ^a^	0.337	0.334	1,738	0.337	101,546	2	399	0.000

^a^Predictors: (Constant), conflicts in the school environment, social exclusion. ^b^Dependent variable: verbal attacks as an aggressor.

The observation that the model explains approximately 33.7% of the variance in verbally aggressive behavior emphasizes that, although social exclusion and school conflicts are influential elements, there are other contributing variables, such as individual traits, family context, reference group influences, or other environmental factors.

The summary of the model shows a moderate but significant connection between social exclusion, school conflicts, and verbal attacks as an aggressor. The results emphasize the importance of analyzing social and environmental factors in understanding and addressing verbally aggressive behavior, while recognizing the contribution of other variables in this behavioral spectrum, which indicates the need for an integrated and exhaustive perspective in research and practice.

To investigate the existence of a significant statistical difference in the frequency of “Verbal attacks as an aggressor,” in the context of the influence of the predictor variables “Conflicts in the school environment” and “Social exclusion,” the analysis of variance (ANOVA) was applied, as presented in Table 7. The significance of the model, evidenced by a *p-*value (Sig. = 0.000) below the 0.05 threshold, confirms the statistical relevance of the model, indicating a significant association between the mentioned variables and the dependent variable.

**Table 7:** ANOVA^a^ analysis of variance, between the dependent variable and the predictor variable.

Model		Sum of squares	df	Mean square	*F*	Sig.
1	Regression	613,528	2	306,764	101,546	0.000 ^b^
	Residual	1,205,350	399	3,021		
	Total	1818,878	401			

^a^Dependent variable: verbal attacks as an aggressor. ^b^Predictors: (Constant), conflicts in the school environment, social exclusion.

In the context of the influence of the predictor variables on the frequency of verbal attacks, the ANOVA results show that the model including the variables “Conflicts in the school environment” and “Social exclusion” explains an important portion of the variation in “Verbal attacks as an aggressor.” This demonstrates that the negative interactions characteristic of the school environment, in the form of conflicts and social exclusion, can have a considerable contribution to the manifestation of verbally aggressive behavior among students.

The evaluation of the coefficients within the regression model, according to the data presented in Table 8, contributes to the elucidation of the degree of influence exerted by each predictive variable on the dependent variable, in this context, “Verbal attacks as an aggressor.”

**Table 8:** Coefficients^a^-the regression coefficient between the dependent variable and the predictor variables.

Model		Unstandardized coefficients		Standardized coefficients	*t*	Sig.	Correlations			Collinearity statistics	
		*B*	Std. error	Beta			Zero-order	Partial	Parthian	Tolerance	VIV
1	(Constant)	3,343	0.233		14,356	0.000					
	Social exclusion	0.294	0.025	0.590	11,736	0.000	0.581	0.507	0.478	0.658	1,521
	Conflicts in the school environment	−0.008	0.027	−0.016	−0.312	0.755	0.329	−0.016	−0.013	0.658	1,521

^a^Dependent variable: verbal attacks as an aggressor.

In the multiple regression, the potential predictors of the bullying behavior of students in the position of aggressors were entered in the ascending order of the correlation coefficients obtained by each of them with the conflicts in the school environment. The regression equation of bullying behaviors in the educational environment has the following elements:

- The adjusted *R*^2^ is 0.33, which means that 33% of the variation in bullying behaviors in the educational environment is explained by the predictors included in the regression model. The rest of the variance, 67% (100–33%), is explained by other variables that were not investigated in the present study (confounding variables). This indicates that there are other variables or factors influencing bullying behaviors that were not measured or included in this study, such as:
- Personal factors, including students’ personality traits, such as impulsivity and empathy,
- Family factors, such as family dynamics, parenting style, and the family’s socio-economic conditions,
- Cultural and socio-economic factors, including cultural norms regarding violence and aggressive behavior, as well as the community’s socio-economic level,
- The influences of the online environment and social networks, such as exposure to cyberbullying and the intensive use of social media platforms.

Other confounding variables could include the effectiveness of anti-bullying programs and school disciplinary policies, interpersonal relationships with peers and teachers, social support from friends and mentors, and the mental and emotional state of the students.

- *F*-test (ANOVA) values and significance coefficients having values less than 0.000 confirm that the model is valid

Knowing the level of aggressive manifestations in the educational environment and aggressive behaviors using the regression equation – *verbal attacks as an aggressor = 3.343 + (0.294) social exclusion – (0.008) conflicts in the school environment* – we obtain the level of bullying behaviors of students from the aggressor position.

The consistent and statistically significant coefficient associated with social exclusion reconfirms the relevance of this variable as the main predictor in the manifestation of verbal attacks. This implies that the phenomena of isolation or social marginalization are catalytic factors of verbally aggressive behavior.

In contrast, the insignificant and reduced coefficient for the variable “Conflicts in the school environment” suggests that, in this specific model and the context of the analyzed data, school conflicts lack a direct and significant influence on verbal attacks, especially compared to the impact exerted by exclusion social. This does not denote the insignificance of school conflicts *per se*, but rather a secondary role in this analytical framework.

The conclusions of the regression model emphasize the significance of social exclusion as an essential determinant in the etiology of verbal attacks as an aggressor. Although the variable “Conflicts in the school environment” did not demonstrate a significant influence in this model, its role in understanding the complexity of aggressive behavior in the educational sphere should not be neglected. Preventive and intervention strategies should recognize and address this complexity, focusing on strengthening social cohesion and improving the school climate to diminish manifestations of verbal aggression and cultivate a conducive and supportive educational environment.

**Figure 2:**
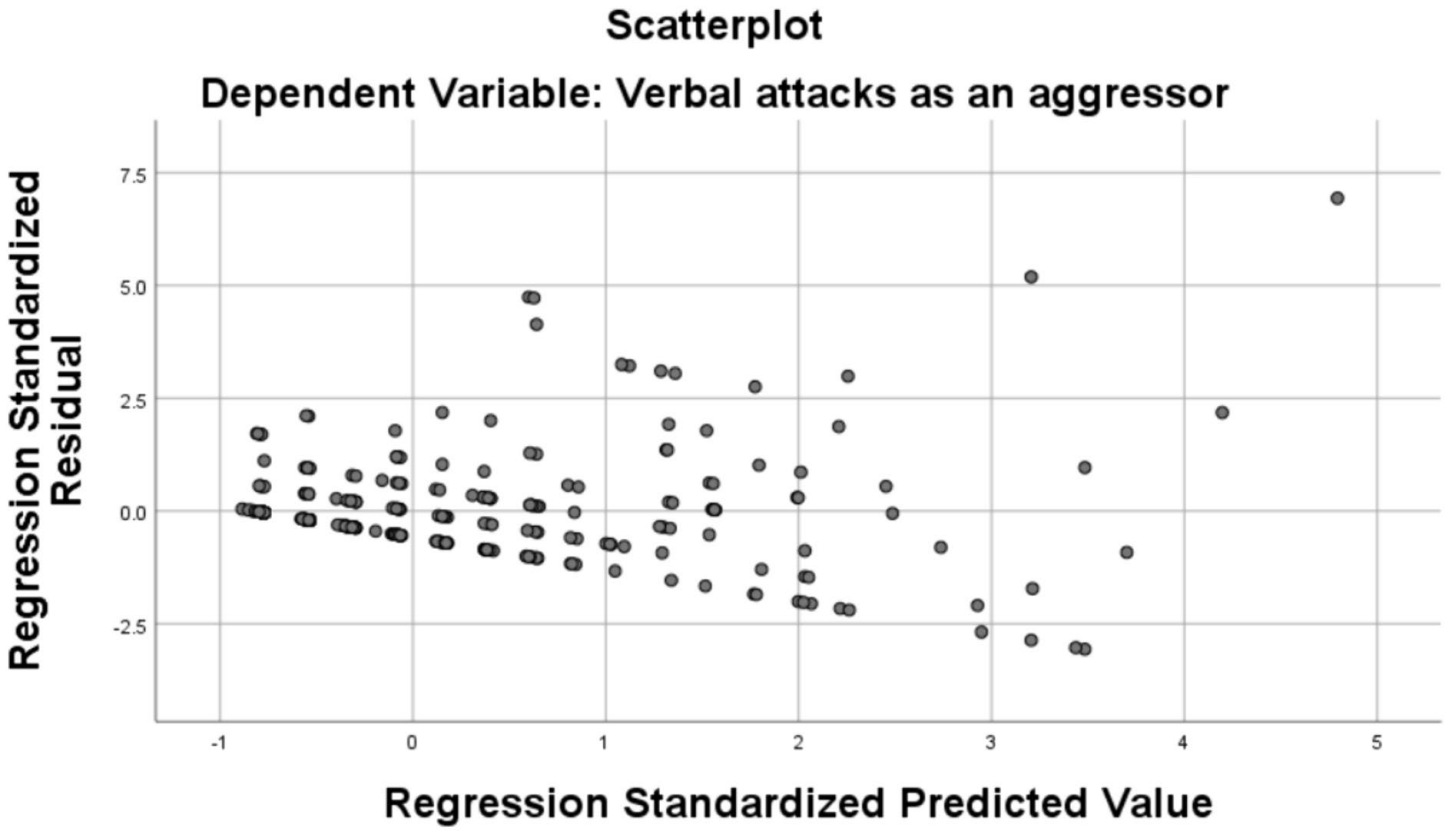
Regression graph.

Figure 2 shows a relatively uniform scatter of the points in the plot, which suggests that the model exhibits the property of homoscedasticity, i.e., it exhibits a constant variance of the residuals over the entire range of predicted values. The absence of a visible pattern of expansion or contraction of the residuals according to the standardized predicted values is a positive indicator and suggests a good fit of the model to the data.

The regression model assumes a linear relationship between the predictor variables and the dependent variable.

The fact that no systematic or skewed pattern is observed in the distribution of points on the plot supports the hypothesis that the assumption of linearity is properly met within this data set.

In conclusion, the graph indicates an adequate performance of the regression model, but also signals the possibility of improvement, especially due to the presence of extreme values (outliers) that can influence the results of the model. For a more comprehensive assessment and to make informed decisions about the model, it would be beneficial to check other regression diagnostics, such as the level of leverage and measures of influence, as well as a detailed analysis of outliers to understand why these observations deviate from the general trend of the model.

Hypothesis 3 (*H3*): The existence of correlations between tolerance toward unethical behaviors and the publication of unauthorized information/violation of privacy are predictors of bullying behavior in students.

Examination of the descriptive statistics provides detailed insight into the data collected regarding aggressive behavioral manifestations and associated predictive factors, such as breach of confidentiality, dissemination of information without authorization, and acceptance of unethical behaviors, in a sample of 401 students.

The average values recorded, especially the average of 6.33 for the variable “Bullying behavior,” indicate a significant prevalence of aggression among students. The standard deviation of 2.056 for this variable reflects moderate variability in responses, suggesting differences in the degree of aggression reported by students.

The average value of 5.39 for the “Violation of privacy” variable highlights the presence of this phenomenon, although at a lower level compared to bullying behavior. A standard deviation of 1.659, less than that associated with bullying behavior, suggests greater consistency in student responses regarding privacy violations.

The mean of 3.50 for the variable “Dissemination of information without authorization” indicates a perception of a low frequency of this type of behavior among students. Conversely, the mean of 5.84 for the variable “Tolerance of unethical behaviors” suggests a moderate to high acceptance of unethical behaviors among students. The standard deviation of 2.381 for this variable indicates diverse opinions among students regarding the acceptability of unethical behaviors.

These values suggest that although bullying behaviors and tolerance of unethical behaviors are reported to be relatively common, the phenomena of privacy violations and unauthorized dissemination of information are perceived as less common. This could reflect a culture where certain forms of bullying and ethical violations are more accepted or overlooked compared to others.

Greater variability in tolerance of unethical behaviors could signal divergence within the school community regarding ethical norms and values. Differences in students’ perceptions of what is ethical or acceptable can influence both individual behavior and collective reactions to bullying by others.

The reduced mean value for disseminating information without authorization could indicate a clearer awareness of privacy and privacy issues in the digital space or the perception of more serious consequences for such actions.

Applying Pearson correlation analysis in this context reveals the magnitude and directionality of linear associations between four distinct variables: “Bullying behavior” “Invasion of privacy as a bully” “Dissemination of information without authorization” and “Acceptance of unethical behaviors” on a sample of 401 cases.

The moderate and positive correlations identified between “Violation of privacy as an aggressor” (0.388) and “Dissemination of information without authorization” (0.412) suggest a concurrent association between these behaviors; that is, the presence of aggressive behavior is frequently associated with the presence of other forms of aggression.

This observation could indicate common personality traits or a school environment that facilitates the manifestation of these behaviors.

The low correlations between “Acceptance of unethical behavior” (0.176) and the other variables involved in the study suggest that permissive attitudes are not necessarily direct predictors of aggressive manifestations. However, even a correlation of low strength can have important meanings in a sample of considerable size.

Except for the relationship between “Acceptance of unethical behaviors” and “Violation of privacy as an aggressor,” all correlations are statistically significant at the 0.000 or 0.002 level (one-tailed test), signaling a minimal probability that these associations are the result of chance.

Thus, the observed correlations highlight interconnections between various types of aggressive behaviors among students, the strongest relationship being between “Violation of privacy as an aggressor” and “Dissemination of information without authorization“. Although the correlation with “Acceptance of unethical behaviors” is significant, the intensity of this association is lower, which suggests that the permissive attitude is not as predictive of aggressive behaviors as the presence of other forms of aggression.

Since the KMO coefficient is 0.685 (Table 9), we can moderately consider that these existing correlations between the studied variables are not due to chance, which allows us to apply factor analysis to determine the percentage of influence of bullying behaviors based on tolerance toward unethical behaviors, privacy violations by the aggressor, and the publication of unauthorized information.

**Table 9:** KMO and Bartlett’s test.

Kaiser-Meyer-Olkin measure of sampling adequacy.		0.685
Bartlett’s test of sphericity	Approx. Chi-Square	233.968
	df	6
	Sig.	0.000

The summary model evaluation, presented in Table 10, explores the dynamics between three predictor variables: “Acceptance of unethical behaviors,” “Violation of privacy as a bully” and “Dissemination of information without authorization” — and the dependent variable “Bullying behavior.” The multiple correlation coefficient *R*, with a value of 0.470, reveals a correlation of moderate intensity between the predictor variables and the dependent variable, meaning that there is a tendency to increase the manifestations of bullying with the intensification of the values of the predictor variables. The coefficient of determination R Square, registering the value of 0.221, illustrates that the model explains approximately 22.1% of the variation in aggressive behavior. This suggests that although the model provides insight into aggressive behaviors, a significant percentage of variance is still not explained. A correlation of moderate intensity and an explanatory proportion of about 22% underlines the fact that attitudes toward unethical behaviors and online manifestations, such as privacy violations and dissemination of information without authorization, exert a recognized influence on bullying behaviors. However, these variables are not the only factors contributing to this behavioral dynamic.

**Table 10:** Model summary–the association between the dependent variable and the predictor variables.

Model	*R*	*R* square	Adjusted *R* square	Std. error of the estimate	Change statistics				
					*R* square change	*F* change	df1	df2	Sig. *F* change
1	0.470^a^	0.221	0.215	1,822	0.221	37,487	3	397	0.000

^a^Predictors: (Constant), tolerance toward unethical behaviors, violation of privacy as aggressor, publication of unauthorized information. ^b^Dependent variable: bullying behavior.

The analysis of variance (ANOVA) study, according to the data in Table 11, is implemented to assess the existence of statistically significant differences between the means of various groups. In the context of regression analysis, ANOVA is used to test whether the proposed regression model that includes the predictor variables is significantly different from a null model—a model that assumes no relationship between the predictor variables and the dependent variable.

**Table 11:** ANOVA^a^- analysis of variance, between the dependent variable and the predictor variable.

Model		Sum of Squares	df	Mean Square	F	Sig.
1	Regression	373,179	3	124,393	37,487	0.000 ^b^
	Residual	1,317,370	397	3,318		
	Total	1,690,549	400			

^a^Dependent variable: aggressive behaviors. ^b^Predictors: (Constant), tolerance toward unethical behaviors, violation of privacy as aggressor, publication of unauthorized information.

The ANOVA results demonstrate that the regression model, integrating the three predictors, contributes significantly to explaining the variation in aggressive behaviors. This observation indicates that the variables “Acceptance of unethical behaviors,” “Violation of privacy as an aggressor” and “Dissemination of information without authorization” have notable importance in decoding the dynamics of bullying behaviors within the examined population.

Table 12 shows the coefficients obtained in the multiple linear regression model, which investigates the impact of three independent variables — “Violation of privacy as an aggressor,” “Dissemination of information without authorization” and “Acceptance of unethical behaviors”— on the dependent variable “Bullying behavior.”

**Table 12:** Coefficients^a^.

Model		Unstandardized Coefficients		Standardized coefficients	*t*	Sig.	Correlations			Collinearity statistics	
		*B*	Std. error	Beta			Zero-order	Partial	Parthian	Tolerance	VIV
1	(Constant)	2,883	0.366		7,884	0.000					
	Violation of privacy as an aggressor	0.291	0.064	0.235	4,557	0.000	0.388	0.223	0.202	0.739	1,352
	Publication of unauthorized information	0.407	0.077	0.276	5,313	0.000	0.412	0.258	0.235	0.727	1,375
	Tolerance toward unethical behaviors	0.078	0.039	0.090	1,993	0.047	0.176	0.100	0.088	0.961	1,040

^a^Dependent variable: bullying behaviors.

In the multiple regression, the potential predictors of bullying behavior in students were entered in the ascending order of the correlation coefficients obtained by each with privacy violation as the aggressor. The regression equation of bullying behaviors in the educational environment has the following elements:

- The adjusted *R*^2^ is 0.221, which means that 22% of the actual cases of bullying in the educational environment are explained by this model, indicating a significant impact on bullying behavior and explaining part of its variance. Variables such as aggressiveness, privacy violations, and unauthorized dissemination of information (as inferred from the previous context) have a significant impact on bullying behavior and can explain part of its variation.
- The remaining 78% of the variance represents the part of bullying behavior variance that is not explained by the variables included in the current model. This highlights the existence of other confounding variables or factors that influence bullying behaviors that were not included in the study, such as:
- Family factors, including family dynamics, parenting style, and the presence of domestic conflicts,
- Socio-economic factors, such as parent’s education and income levels and the community’s economic conditions,
- Online environment influences, including exposure to cyberbullying and the use of social networks,
- Cultural factors, referring to cultural and social norms regarding violence and aggression,
- Individual personality traits, such as empathy and impulsivity,
- Aspects of the school environment, including school culture, anti-bullying policies, and teacher-student relationships,
- Peer group influences, such as peer pressure and belonging to social groups,
- Psychological factors, such as students’ mental and emotional states and the presence of psychological disorders.

*F*-test (ANOVA) values and significance coefficients having values less than 0.000 confirm that the model is valid.

Knowing the level of aggressive manifestations in the educational environment and aggressive behaviors using the regression equation – *bullying behaviors = 2.883 + (0.291) violation of privacy as an aggressor + (0.407) publication of unauthorized information + (0.078) tolerance toward unethical behaviors* – we obtain the level bullying behaviors of students in the educational environment.

In the analyzed model, the coefficient B of 0.291 indicates that a unit increase in “Violation of privacy as an aggressor” predicts an average increase of 0.291 units in bullying manifestations. This suggests a close association between privacy-violating behaviors and general aggression, highlighting the need to address respect for privacy in anti-bullying interventions.

The B-coefficient of 0.407 indicates that a unit increase in “Dissemination of information without authorization” is correlated with an average increase of 0.407 units in bullying behavior. This observation suggests that the influence of unauthorized dissemination of information on bullying is even more pronounced than that of invasion of privacy. This strong predictor could indicate a direct connection between disregard for the privacy of others and the propensity for aggressive behavior, thus directing educational programs to emphasize understanding of the repercussions of online activities.

The coefficient B of 0.078 reflects the fact that a unit increase in “Acceptance of unethical behaviors” is associated with an average increase of 0.078 units in bullying manifestations. Although this effect is smaller compared to the other two variables, a positive influence persists. This underlines the fact that permissive attitudes toward unethical behaviors can foster an environment conducive to aggressive manifestations.

In summary, the three variables—violation of privacy, unauthorized dissemination of information, and acceptance of unethical behaviors—are found to be significant predictors of bullying behavior. However, disseminating information without authorization stands out as having the most pronounced impact, followed by invasion of privacy, while accepting unethical behaviors has the least impact.

**Figure 3:**
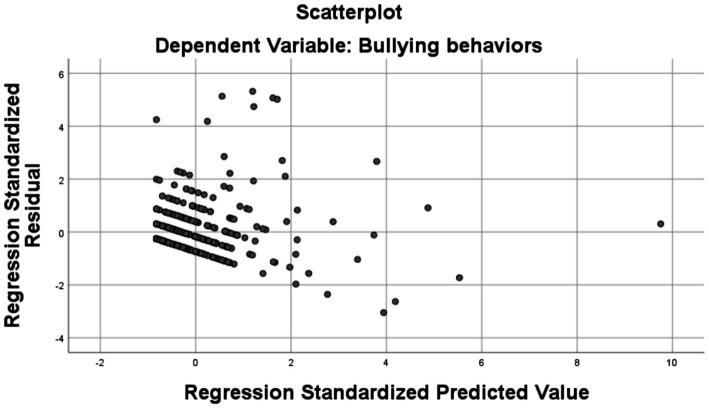
The regression scatterplot.

Figure 3 illustrates a scatterplot of the standardized residuals compared to the standardized predicted values obtained from a regression model with “Bullying Behavior” as the dependent variable.

The pronounced residuals could reflect situations where the model failed to adequately capture student bullying behavior, possibly due to the omission of relevant factors from the analysis. An increase in the variance of the residuals consistent with the predicted values may signal a reduced fit of the model for subjects with more pronounced aggressive manifestations, which could be attributed to complex elements governing these behaviors.

Students associated with extreme values (outliers) could be characterized by particular circumstances or face a distinct social dynamic, which predisposes them to bullying behaviors.

Exploring and interpreting these cases could provide valuable insights essential for designing educational interventions.

The graph indicates a moderate ability of the regression model to predict bulling behavior, while also signaling the possibility of optimization of the model. This could involve the inclusion of new predictor variables or a deeper analysis of atypical cases. From the perspective of educational psychology, these findings can be applied to the development and implementation of more effective anti-violence programs, adapted to the diversity of students’ experiences and behaviors.

## 5 Discussions and conclusion

The analysis of bullying behaviors among adolescents, in the context of this study, reflects the complex interaction between individual and environmental factors. The results obtained emphasize the significant role of aggression, violation of privacy, and unauthorized dissemination of information, in line with the existing literature that identifies these aspects as relevant to bullying manifestations (Li et al., 2024; Del Moral et al., 2019).

The finding that social exclusion and conflicts in the school environment significantly contribute to the escalation of bullying behaviors reiterates the importance of creating an inclusive and safe school environment. These findings are consistent with previous research emphasizing the need to proactively address conflict and promote social integration to reduce the prevalence of bullying (Schuetz et al., 2022; Saarento et al., 2015).

Interestingly, although the acceptance of unethical behaviors seems to have a lower impact compared to the previously mentioned variables, this aspect does not diminish the importance of cultivating a robust ethical framework among students. This result suggests that although attitudes toward unethical behaviors are not direct predictors of bullying behaviors, they contribute to the general climate that may favor or discourage such manifestations.

The results also indicate a significant association between aggressive behaviors and the dissemination of information without authorization, underscoring the importance of education on the responsible use of technology. This aspect is essential in today’s context, where technology plays an increasing role in the lives of teenagers.

It is important to note that this study has specific limitations, being a cross-sectional rather than a longitudinal research. This limits our ability to establish causal relationships between variables and to observe the evolution of bullying behaviors over time. Data were collected at a single point in time, which may affect the interpretation and generalization of the results.

In the context of this study, it was noted that interventions should target both the aggressive behaviors themselves and the contextual factors that may influence them. Promoting socio-emotional skills and empathy, along with encouraging ethical behavior, are essential to address the phenomenon of bullying effectively.

The present study contributes to the existing literature by exploring in detail the predictors of bullying among adolescents, providing an integrated perspective on the interaction between individual characteristics and the socio-school context. The obtained results underline a complex set of variables that influence bullying behavior, among which aggression, violation of privacy, and dissemination of information without authorization stand out as having a significant impact.

The current study extends the literature by investigating the predictive factors of bullying among adolescents, providing an integrated analysis of the interactions between personal characteristics and the socio-school context (Gómez-Ortiz et al., 2019).

The finding that social factors, such as social exclusion and conflicts in the school environment, exacerbate the manifestations of bullying highlights the importance of building an educational environment based on inclusion and effective conflict management. These findings reiterate the need for a multidisciplinary approach to the prevention and intervention against bullying, involving not only formal education but also the development of socio-emotional skills and the promotion of a positive school culture.

The study results highlight a complex array of variables that influence bullying behavior. Notably, aggression, invasion of privacy, and unauthorized dissemination of information are noted to have a substantial impact (Clarke and Kiselica, 1997).

Previous studies confirm that social factors, such as social exclusion and school conflicts, amplify the manifestations of bullying, thus emphasizing the need for an inclusive and effective educational environment in conflict management (Johnson and Smith, 2021).

At the same time, the influence of technology on bullying behaviors, especially the violation of privacy and the dissemination of information without authorization, underlines the importance of digital education among adolescents. This suggests that prevention programs should include components that encourage responsible use of technology and promote awareness of the consequences of online actions.

The study identified the significant influence of technology on bullying behaviors, particularly in the areas of invasion of privacy and dissemination of information without consent. These findings are consistent with research that emphasizes the importance of digital education for adolescents (Miller and Connelly, 2021).

Although the study identified a relatively smaller influence of tolerance toward unethical behaviors on bullying, this aspect should not be neglected in the development of effective interventions. Promoting a strong moral framework and encouraging ethical behaviors can help create a school environment where bullying is explicitly discouraged.

Although a reduced influence of tolerance toward unethical behaviors on bullying was observed, this aspect remains crucial in the development of effective interventions. Interventions must be personalized and tailored to the specific needs of school communities, an aspect supported by the need for collaboration between educators, parents, and students to ensure a safe and supportive environment (Williams et al., 2022).

At the same time, interventions must be personalized and adapted to the specific needs of school communities, considering the variety of factors that contribute to the phenomenon of bullying. Collaboration between educators, parents, and students is essential to the successful implementation of these strategies, thereby ensuring a safe and supportive environment for all adolescents.

The present study has theoretical implications, among which we mention:

The study can contribute to the expansion of existing theories about aggression, through the specific contextualization of cultural, social, and economic factors in Romania that influence bullying behavior. This can lead to adaptations of aggression theories that take into account specific local and regional variables.

The results of the study can validate the effectiveness of the socio-emotional competence model in the Romanian context, indicating the need to adapt educational programs to include specific components that address aggression and bullying behaviors.

By identifying the link between social exclusion and bullying, the study adds an important dimension to theories of social inclusion. This suggests that effective interventions must promote better social integration in schools as a strategy to prevent bullying.

We also highlight the practical implications, based on the results of the study, schools in Romania could develop and implement educational programs that incorporate education for socio-emotional skills, with an emphasis on empathy, anger management, and resilience. These programs can be integrated into the national curriculum as preventive measures against bullying.

Another practical implication refers to the involvement of teachers in recognizing early signs of bullying behavior and appropriate intervention is crucial.

Professional training should include specific modules on early intervention strategies and conflict management, adapted to the cultural and social context in Romania.

The implementation of clear and strict school policies on bullying can deter bullying behaviors and stabilize a safe and inclusive school environment, which can be seen as a third practical implication of the study. These policies should be well communicated to all members of the school community, from students to parents and school staff.

These theoretical and practical implications underline the importance of an integrated and well-grounded approach to combating bullying in Romanian schools, with potential benefits both at the individual level and at the level of the entire educational community.

Recommendations for future research include adopting a longitudinal design to observe the evolution of bullying behaviors and associated factors over an extended period, thus providing a deeper understanding of causal relationships. It is necessary to expand the sample to include a wider variety of schools and regions, thereby enhancing the representativeness and generalizability of the obtained results.

Integrating additional variables, such as the influence of the family environment and the impact of media, could provide a more complete perspective on the factors contributing to bullying behaviors.

Additionally, investigating gender differences in the manifestations and perceptions of bullying is essential for developing tailored interventions that address the specific needs of boys and girls.

In conclusion, our study adds a valuable contribution to understanding the bullying phenomenon, providing clear recommendations for future research and practical applications.

The continuation of research in this field is essential for the adaptation and constant improvement of scientific approaches in the prevention and intervention against bullying, with the ultimate goal of ensuring adolescents’ well-being and positive development in healthy and inclusive educational environments (Harris and Jones, 2019).

### 5.1 Limitations of the study

Like any other research, the present study involves certain limitations. Firstly, the study was cross-sectional, with all instruments used in the study being completed at a single time point. Future studies could be conducted in multiple waves so that causal inferences can be drawn about the investigated relationships.

Secondly, all studied variables were measured with self-report questionnaires. In future research, performance could be measured based on objective indicators.

Thirdly, the study was cross-sectional, therefore no causal conclusions can be drawn. The investigated relationships may have meaning in several directions. Future research could use longitudinal designs in which data are collected at multiple time intervals to estimate the causal order of the investigated relationships.

The unexplained variance in this study suggests that there are a significant number of additional variables influencing bullying behaviors that were not included in this study. This underscores the need to extend the research to identify and measure these confounding variables in a longitudinal study so that more precise regression models can be developed and more effective interventions and policies for preventing and managing bullying in the educational environment can be created.

We intend to transform this study into a longitudinal one by conducting annual measurements on the studied cohort. This approach will allow for the comparison of results over time and an in-depth observation of the stability of bullying behaviors.

A longitudinal study offers a detailed perspective on the evolution and persistence of bullying behaviors among students. By collecting annual data, we will be able to analyze changes and constants in participants’ behaviors, providing a clearer understanding of the dynamics of bullying in an educational context.

Repeated measurements on the same cohort will allow for the observation of changes in behavior and the assessment of the long-term impact of contextual and individual variables. We will collect annual data on bullying incidents, including verbal attacks, and correlate these data with factors such as social exclusion, school conflicts, academic performance, and psychosocial health.

Longitudinal analysis will detect patterns of stability or change in bullying behaviors, providing a solid basis for recommendations on educational policies and intervention strategies. The results obtained will contribute to a deeper understanding of the bullying phenomenon and support the development of better-founded prevention and intervention programs.

## Data Availability

The raw data supporting the conclusions of this article will be made available by the authors, without undue reservation.
